# Blood amyloid-β oligomerization associated with neurodegeneration of Alzheimer’s disease

**DOI:** 10.1186/s13195-019-0499-7

**Published:** 2019-05-10

**Authors:** Young Chul Youn, Sungmin Kang, Jeewon Suh, Young Ho Park, Min Ju Kang, Jung-Min Pyun, Seong Hye Choi, Jee Hyang Jeong, Kyung Won Park, Ho-Won Lee, Seong Soo A. An, Jacqueline C. Dominguez, SangYun Kim

**Affiliations:** 1 Department of Neurology, Chung-Ang University College of Medicine, Seoul, Republic of Korea; 2 Research and Development, PeopleBio Inc., Gyeonggi-do, Republic of Korea; 3 Department of Neurology, Seoul National University College of Medicine, Seoul National University Bundang Hospital, Gyeonggi-do, Republic of Korea; 4 Department of Neurology, Veterans Health Service Medical Center, Seoul, Republic of Korea; 5 Department of Neurology, Inha University School of Medicine, Incheon, Republic of Korea; 6 Department of Neurology, Ewha Womans University Mokdong Hospital, Seoul, Republic of Korea; 7 Department of Neurology, Dong-A University College of Medicine and Institute of Convergence Bio-Health, Busan, Republic of Korea; 8 Department of Neurology, Kyungpook National University School of Medicine, Daegu, Republic of Korea; 9 Department of Bionanotechnology, Gachon University, Incheon, Republic of Korea; 10 Institute for Neurosciences, St. Luke`s Medical Center, Quezon City, Philippines

**Keywords:** Oligomerization, Blood-based biomarker, Amyloid β, Oligomer, Multimer detection system, Voxel-based morphometry, Multimer detection system-oligomeric Aβ

## Abstract

**Introduction:**

Oligomeric amyloid-ß is a major toxic species associated with Alzheimer’s disease pathogenesis. Methods used to measure oligomeric amyloid-β in the blood have increased in number in recent years. The Multimer Detection System-Oligomeric Amyloid-β (MDS-OAβ) is a specific method to measure oligomerization tendencies in the blood. The objective of this study was to determine the association between amyloid-ß oligomerization in the plasma and structural changes of the brain.

**Methods:**

We studied 162 subjects composed of 92 community-based normal healthy subjects, 17 with subjective cognitive decline, 14 with mild cognitive impairment and 39 with Alzheimer’s disease dementia. All subjects underwent MDS-OAβ and three-dimensional T1 magnetic resonance imaging. To determine the structural changes of the brain that are statistically correlated with MDS-OAβ level, we used voxel-based morphometry with corrections for age and total intracranial volume covariates.

**Results:**

We found brain volume reduction in the bilateral temporal, amygdala, parahippocampal and lower parietal lobe and left cingulate and precuneus regions (family-wise error, *p* < 0.05). Reduction was also found in white matter in proximity to the left temporal and bilateral lower parietal lobes and posterior corpus callosum (family-wise error, *p* < 0.05). Brain volume increment was not observed in any regions within grey or white matter.

**Discussion:**

Findings suggest that substantial correlation exists between amyloid ß oligomerization in the blood and brain volume reduction in the form of Alzheimer’s disease despite of uncertainty in the casual relationship.

**Electronic supplementary material:**

The online version of this article (10.1186/s13195-019-0499-7) contains supplementary material, which is available to authorized users.

## Introduction

Biomarkers play an important role in the diagnosis, staging and the risk assessment of disease. Especially, in clinical trials of disease-modifying drugs for Alzheimer’s disease (AD), the biomarker is necessary in selecting subjects and predicting responders to a specific drug intervention and monitoring the responsiveness. Extracellular accumulation of amyloid-β (Aβ) plaques and intracellular neurofibrillary tangles is the characteristic feature of AD [1–3]. Aβ is produced by cleavage of amyloid precursor proteins into a monomeric form by β-secretase and γ-secretase, which is transformed into oligomeric form and fibre form, and finally into amyloid plaques [4]. Among them, oligomeric Aβ is the major toxic species of Aβ associated with AD pathology as well as synaptic dysfunction [5, 6], and this could be the final target of AD biomarker. Currently validated amyloid biomarkers of AD are cerebrospinal fluid Aβ1–42 level and amyloid positron emission tomography imaging [7]. However, there are some limitations, such as invasiveness, interlaboratory variability, and cost. Many researchers have focused on the identification of blood-based biomarkers for AD but, up to the present, no biomarker having a direct association with the pathogenesis of AD has been found [8, 9]. Recently, new methods, advanced in terms of invasiveness and cost and aimed at measuring oligomeric Aß in the blood, emerged in numbers.

The Multimer Detection System-Oligomeric Aβ (MDS-OAβ) is one of these promising methods for differentiating AD measuring the oligomerization tendency of spiked synthetic Aβ in the plasma [10]. This basically utilizes Multimer Detection System, a sandwich assay that uses capture antibodies and epitope-overlapping detection antibodies to preferentially detect oligomers or multimers from monomers in protein-misfolding diseases through competitive bindings to specific epitopes [11–13]. MDS-OAβ basically exploits the Aß dynamic changes observed in the blood of AD to measure the oligomerization tendency. Previous reports have demonstrated the value of MDS-OAβ in AD diagnosis by comparing it with other biomarkers, cerebrospinal fluid (CSF) biomarkers and amyloid positron emission tomography images but did not show that MDS-OAβ reflects the pathological mechanism of AD [11]. In this study, we hypothesized that higher Aß oligomerization in the blood are associated with the neuronal degeneration of the brain in the form of AD. Many recent studies have utilized voxel-based morphology (VBM), an automated volumetric analysis procedure. VBM has the advantage of being a relatively faster analysis process and generating consistent results [14, 15]. Therefore, it was anticipated that this VBM study would demonstrate correlation between volume changes in cortical grey matter (GM) and white matter (WM) and Aß oligomerization as measured by MDS-OAß and, furthermore, relate to atrophic change of AD brain [16, 17].

## Methods

This study was based on data obtained from one of the ‘Dementia Overcoming Projects in Korea’—a study for the development of protein biomarkers and clinical studies for the early diagnosis of dementia, which has been funded by the Ministry of Health & Welfare since 2015. From May 2015 to December 2017, a total of 188 ‘cognitive normal’ subjects, 38 subjects with ‘mild cognitive impairment (MCI)’, and 202 subjects with ‘AD’ were recruited in 5 disorder clinics at university-based hospitals. A subset of this group was randomly selected and subjected to MDS-OAβ. We analysed MRI using voxel-based morphometry (VBM) to locate the regions within cortical grey matter (GM) and white matter (WM) where volume changes correlated with MDS-OAβ levels.

### Subjects

Aβ oligomerization tendency in the plasma of 187 subjects was measured using MDS-OAβ; however, 25 subjects were not suitable for analysis as no three-dimensional T1-weighted magnetic resonance imaging was performed. Accordingly, 162 subjects were eligible for this study. The images of 12 subjects could not be converted from the raw Digital Imaging and Communications in Medicine (DICOM) format into the Neuroimaging Informatics Technology Initiative (nifti) format. Further, 10 subjects dropped out during pre-processing due to errors, and 3 others dropped out after quality check.

**Fig. 1:**
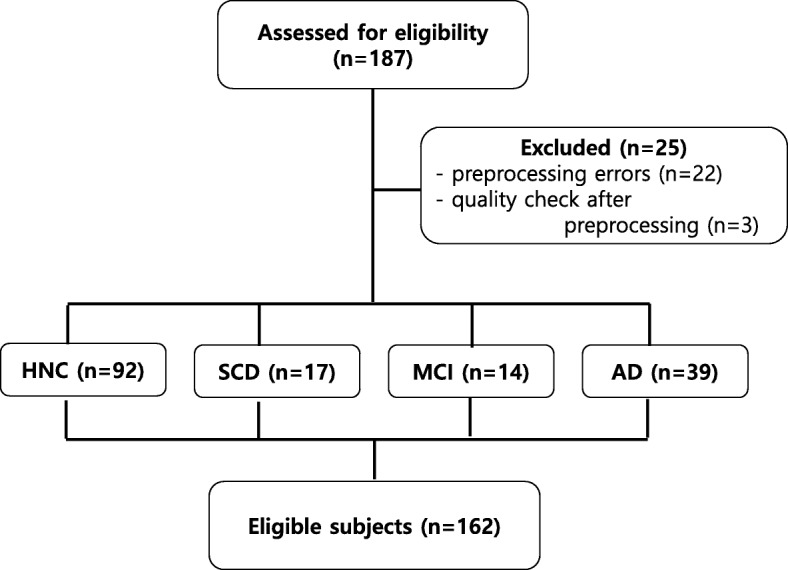
Enrolment of eligible subjects

The three-dimensional T1-weighted magnetic resonance imaging data of 162 subjects were from 92 community-based healthy normal controls (HNC), 38 with AD, 15 with mild cognitive impairment due to AD, and 17 with subjective cognitive decline (SCD) (Fig. 1).

The inclusion criteria for HNC in the present study were as follows: (1) the absence of memory complaints, (2) normal general cognition (within 1 standard deviation (SD) of the age- and education-adjusted norms of the Korean version of the MMSE and a score > 26), (3) intact activities of daily living (ADL), (4) Korean Dementia Screening Questionnaire < 7 [18] and (5) the absence of depression (short form geriatric depression score < 8). The following comprise those for SCD: (1) the presence of memory complaints; (2) normal general cognition (within 1 standard deviation (SD) of the age- and education-adjusted norms of the Korean version of the MMSE (K-MMSE) [19], and a score of > 26); (3) intact performance of daily living (ADL); and (4) no abnormality on a comprehensive neuropsychological battery (within 1 SD of age- and education-adjusted norms) [20]. The criteria for MCI were as follows: (1) the presence of memory complaints; (2) intact performance of ADL; (3) objective cognitive impairment in more than one cognitive domain on a comprehensive neuropsychological battery (at least 1.0 SD below age- and education-adjusted norms); (4) CDR of 0.5; and (5) not demented according to the Diagnostic and Statistical Manual of Mental Disorders (DSM)-IV criteria. The patients with AD dementia (ADD) met the probable AD criteria proposed by the National Institute of Neurological and Communicative Disorders and Stroke and AD and Related Disorders Association (NINCDS-ADRDA) [21], as well those proposed by the DSM-IV.

#### The measurement of oligomerization of Aβ in plasma

MDS-OAβ was used to measure OAβ [12, 13]. For this method, epitope-overlapping antibodies specific for the N-terminus of Aβ were used to capture and detect the Aβ antigen in its multimeric or oligomeric form. The mouse monoclonal antibody 6E10 (BioLegend, San Diego, CA, USA) and WO2-HRP antibody (Absolute Antibody Ltd., Oxford, UK) were chosen to detect OAβ in MDS due to their sensitivity and specificity. The epitopes for these antibodies overlap at the N-terminus of Aβ. To utilize MDS, the wells of a 96-well black plate were coated with 6E10 antibody at a dilution of 3 μg/ml in carbonate-bicarbonate buffer (Sigma-Aldrich, St. Louis, MO, USA) and incubated overnight at 4 °C (Thermo Fisher Scientific, Waltham, MA, USA). The plates were blocked for 2 h with 0.4% Block Ace (100 μl) at RT. After washing three times with phosphate-buffered saline (PBS; Sigma-Aldrich, St. Louis, MO, USA), the plate was stored at 4 °C until use. Prior to the assay, aliquots of plasma samples were thawed at 37 °C for 15 min. A solution containing 10 μl of plasma, 4 μl of HBR-1, a HAMA blocker (Scantibodies Laboratory, Santee, CA, USA), and PBST was mixed. We spiked the synthetic Aβ into plasma mixture and incubated it at 37 °C for 1 h. The plasma sample mixture and serially diluted standards were added to each well of the plate in a total volume of 100 μl. The plates were incubated at RT for 1 h. After washing three times with TBST, the WO2-HRP antibody in TBST containing 0.4% Block Ace was added to the wells, and the plate was incubated for 1 h at RT. Finally, 100 μl of 3,3′,5,5′-tetramethylbenzidine (TMB) reagents (Sigma-Aldrich, St. Louis, MO, USA) was added as a substrate, and the reaction was stopped with 50 μl of 1 M H_2_SO_4_. Optical density (OD) values were measured at 450 nm using a Victor 3TM multi-spectrophotometer.

#### Pre-processing of MRI for VBM analysis

MRI scans were acquired from 3-T scanners manufactured by Philips (Achieva, Amsterdam, the Netherlands) with the DICOM format. The data were analysed using VBM analysis using the Computational Anatomy Toolbox (CAT12) via the current version of Statistical Parametric Mapping software (SPM12). CAT12 is a VBM toolbox implemented in Statistical Parametric Mapping software [22] for performing comprehensive VBM analyses of brain structures, designed by The Structural Brain Mapping Group at the University of Jena (Jena, Germany) for automatic and easy-to-use application [23].

First, we converted the raw DICOM files into nifti format, using MRICRON software (http://people.cas.sc.edu/rorden/mricron/index.html). VBM analyses were conducted using SPM12, and pre-processing was performed through the CAT12 toolbox with the default settings and ‘ICBM template-East Asian Brains’ template. All scans were normalized using an affine followed by non-linear registration, corrected for bias field in homogeneities, and then segmented into GM, WM and CSF components [24]. We used the Diffeomorphic Anatomic Registration Through Exponentiated Lie algebra algorithm (DARTEL) to normalize the segmented scans into a standard Montreal Neurological Institute space [25]. The modulation process was performed, which corrects individual differences in the brain size, consisted of a non-linear deformation performed on the normalized segmented images [26]. We reviewed the GM and WM morphological abnormalities and applied smoothing processes to all segmented, modulated and normalized GM and WM images using 8-mm full-width-half-maximum Gaussian smoothing.

#### Statistical analyses

The smoothed images fed into a linear multiple regression of SPM12 to detect negative correlations between the GM area and the level measured by MDS-OAβ. In CAT12 toolboxes, the total intracranial volumes could be obtained. We used the subject’s age and total intracranial volumes in the matrix design. The GM and WM morphological differences are shown after using a family-wise error (FWE) with a *p* value < 0.05. The extent threshold was set at 50 voxels.

Comparisons between MDS-OAβ levels and ages of the groups and sex were made using the R package (R version 3.5.1) and its library (Rcmdr 2.5-2).

## Results

### MDS-OAβ level of the subjects

The mean age of the subjects was 63.0 ± 9.3 (mean ± standard deviation) years, and 98 were female and 64 male. There was no gender difference according to the clinical states from HNC to AD (Pearson’s chi-squared test, *p* = 0.719). The overall MDS-OAβ levels were 0.903 ± 0.131, and there was no difference between the gender (Welch’s two sample *t* test, *p* = 0.864). It showed tendency to increase from HNC to SCD, MCI and AD (ANOVA, *p* < 0.001), and there was a significant difference from MCI and AD compared to HNC level, but there were age differences between them (Tukey multiple comparison of means, *p* < 0.01) (Table 1).

**Table 1:** Demographics of the subjects

	Subject number (female to male)	MDS-OAβ	Age
Sex			
Female	98	0.904 ± 0.130	63.1 ± 9.6
Male	64	0.901 ± 0.134	62.9 ± 8.8
Clinical state			
HNC	92 (53:39)	0.852 ± 0.130	60.5 ± 7.5
SCD	17(10:7)	0.925 ± 0.111	66.1 ± 9.5
MCI	15(9:6)	0.964 ± 0.098**	70.0 ± 9.2**
AD	38(26:12)	0.993 ± 0.089***	65.1 ± 11.1*

*HNC* healthy normal control, *SCD* subjective cognitive decline, *MCI* mild cognitive impairment; *AD* Alzheimer’s disease dementia

*p value < 0.05, **p value < 0.01, ***p value < 0.001, a significance of the difference when compared with Healthy Normal Control by ANOVA using R package

### VBM analyses of the GM and WM

**Fig. 2:**
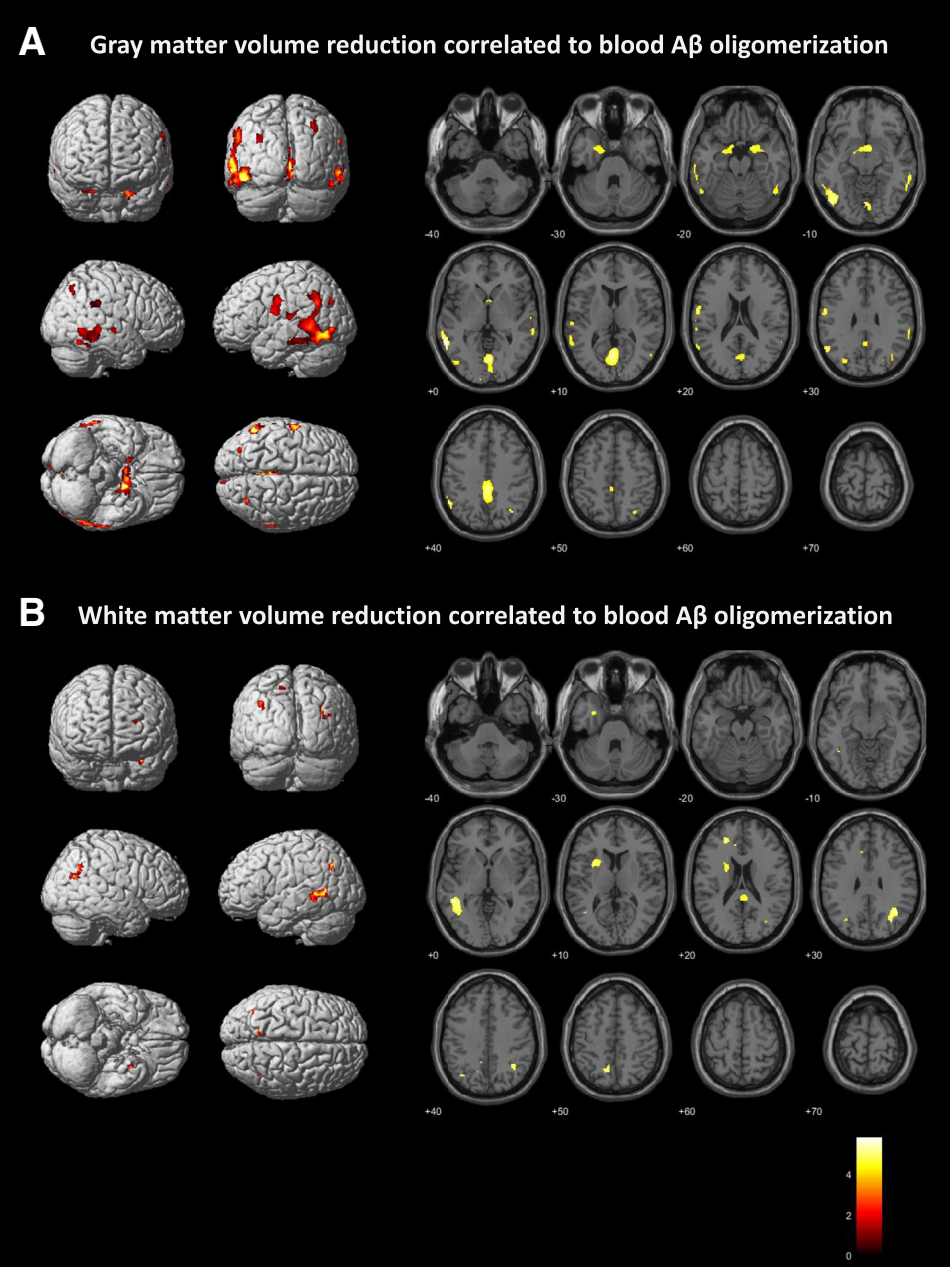
Grey matter (**a**) and white matter (**b**) volume reduction correlated with Aβ oligomerization measured with MDS-OAβ (*n* = 162, corrected for age and total intracranial volume, family-wise error (FWE) < 0.05)

Table 2 and Fig. 2 show significant GM and WM volume change in relation to the oligomerization level measured by MDS-OAß and VBM analysis in all spectrum of subjects which includes HNC, SCD, MCI and AD dementia. Table 2 presents anatomic labelling of brain Montreal Neurological Institute coordinates where reduced volume correlated to Aβ oligomerization in the blood presented in Fig. 2. The areas are statistically significant with correction of multiple comparison problems by FWE. The VBM analysis conducted using CAT12 revealed a significant reduction in GM volume of the bilateral temporal, amygdala, parahippocampal and lower parietal lobe and left cingulate and precuneus (FWE < 0.05) (Fig. 2a). We also found WM volume reduction nearby the left temporal and bilateral lower parietal lobes and posterior corpus callosum (FWE, *p* < 0.05) (Fig. 2b). There was not any location which showed increments of the brain volume related to MDS-OAβ levels.

**Table 2:** Anatomic labelling of Montreal Neurological Institute (MNI) coordinates, maximal intensity and *p* value in voxel level correlated to blood Aβ oligomerization

MNI coordinates			Labels	*t* value	*z* value	*p* value (FWE-corr)
*x*	*y*	*z*				
− 60	− 51	2	Temporal_Mid_L	6.217	5.871	0.000
− 56	− 57	39	Parietal_Inf_L	5.925	5.621	0.000
− 50	− 60	− 9	Temporal_Inf_L	5.769	5.487	0.000
3	− 77	− 8	Lingual_R	5.881	5.583	0.000
0	− 63	11	Calcarine_L	5.731	5.454	0.000
− 2	− 72	9	Calcarine_L	5.614	5.352	0.001
0	− 38	39	Cingulum_Mid_L	5.609	5.348	0.001
0	− 48	38	Precuneus_L	5.435	5.196	0.002
− 3	− 36	50	Cingulum_Mid_L	4.879	4.702	0.015
− 9	− 2	− 15	Amygdala_L	5.449	5.208	0.002
5	2	− 11	Olfactory_L	5.314	5.089	0.003
− 14	− 3	− 26	ParaHippocampal_L	5.028	4.835	0.009
65	− 41	− 12	Temporal_Mid_R	5.334	5.107	0.002
60	− 53	− 6	Temporal_Mid_R	5.236	5.020	0.004
− 59	− 8	35	Postcentral_L	5.289	5.067	0.003
− 60	− 5	21	Postcentral_L	5.265	5.046	0.003
21	2	− 20	ParaHippocampal_R	5.036	4.842	0.008
33	− 2	− 21	Amygdala_R	4.777	4.609	0.023
32	2	− 29	Amygdala_R	4.697	4.537	0.030
− 62	− 23	14	Rolandic_Oper_L	5.006	4.815	0.009
− 62	− 32	18	Temporal_Sup_L	4.753	4.588	0.025
51	− 59	− 18	Temporal_Inf_R	5.002	4.812	0.009
54	− 47	− 23	Temporal_Inf_R	4.680	4.522	0.032
63	− 35	30	SupraMarginal_R	5.000	4.810	0.010
63	− 44	29	SupraMarginal_R	4.737	4.574	0.026
33	− 66	44	Angular_R	4.965	4.778	0.011
33	− 65	53	Parietal_Sup_R	4.633	4.480	0.038
63	− 15	− 5	Temporal_Sup_R	4.901	4.721	0.014
− 32	− 75	35	Occipital_Mid_L	4.788	4.619	0.022

*L* left, *R* right, *Sup* superior, *Mid* middle, *Inf* inferior, *Oper* operculum

## Discussion

In this study, we found that blood MDS-OAβ levels had a negative correlation with brain volume changes of specific regions; locations which showed significant volume reduction were the bilateral hippocampus, posterior cingulate and temporoparietal areas. These are typical areas of degeneration in AD [27, 28], suggesting that the oligomerization of Aβ in the blood can cause a pattern of AD in the brain. We also did VBM analysis of the AD group and found the GM volume reduction in bilateral medial temporal, bilateral dorsolateral temporoparietal and medial parietal areas. These regions are well known for typical sites of degenerative changes in AD (Additional file 1: Figure S1) and coincide with the sites showing volume reduction correlating with MDS-OAß levels. As shown in Table 1 and Additional file 2: Figure S2, MDS-OAß levels may have been affected by the age. However, as the design and objective of the study reside in locating the region of atrophy of the brain in relation to increasing MDS-OAß level as well as the region in the brain where AD pathology occurs, the subjects’ variables were not adjusted before image analysis.

The level of MDS-OAβ may indicate the dynamics of Aβ in the blood. When Aß oligomerization is measured after synthetic Aß is spiked into plasma and incubated over time, the oligomerization dynamics differ between AD and normal healthy subjects [10]. The characteristics of Aβ in the blood are related to changes in brain volume. As far as we know, this is the first study to show that oligomerization tendencies in blood are related to brain volume reductions and that this change is very similar to the brain atrophy patterns demonstrated in AD. This correlation may further support the notion that peripheral blood characteristics could relate to the central brain pathology of AD. Previous work using a transgenic mouse model has also shown that blood-derived Aβ induced AD pathology. The authors demonstrated that Aβ from the AD transgenic mouse brain, which entered the peripheral circulation of wild-type mice after parabiosis surgery, had been transported into the wild-type brain and accumulated over time to form Aβ plaques [29].

When diagnosing AD clinically, cerebrospinal fluid Aβ and amyloid positron emission tomography imaging are widely used and strong methods for identifying amyloidopathy in the brain. However, these methods have limitations, such as invasiveness, inter-laboratory variability and high cost [30].

Blood-based biomarkers, with advantages in terms of low invasiveness and low cost, can serve as an alternative for AD assessment. Validated blood-based biomarkers could be helpful in the diagnosis, monitoring of patients and subject screening in clinical trials [30–32]. Because of these advantages, most clinicians prefer blood-based biomarkers. However, plasma levels of proteins associated with AD are much lower than CSF, and this makes accurate and reliable measurements difficult when conventional enzyme-linked immunosorbent assays are used. Several sensitive methods have been proposed. The ABtest is one of these methods of measuring free and total Aβ 42/40 plasma ratios, currently being used in a research capacity [33]. The SIMOA technology can also quantify lower levels of plasma molecules, such as p-tau181 and Aβ using ultrasensitive immunoassay [34, 35]. Immunomagnetic reduction (IMR) assays also detect the molecules in plasma [36]. Measuring plasma Aβ, tau and p-tau using these techniques would be promising in distinguishing AD from those with subjective cognitive decline, but equipment is required [34, 37]. Another approach is MDS-OAβ, which measures the Aβ oligomerization instead of measuring Aβ species in plasma [10]. It has been shown to correlate well with conventional AD biomarkers such as CSF Aβ42 [11], C-Pittsburgh compound B positron emission tomography standardized uptake value ratio, CSF phosphorylated tau and CSF total tau [11]. Unlike other test methods, it measures Aβ oligomers. With accumulating evidences revealing soluble Aβ oligomers as the major toxic agent in AD pathology, researchers have been trying to develop a way to measure Aβ oligomers in the blood. We used this method to measure the Aβ oligomerization in blood and its correlation with brain atrophy.

We also observed volume changes in WM as well as in GM. WM volume reduction was also observed near GM volume reduction areas, the left temporal lobe, the lower parietal lobe and the insula area. WM volume changes in Alzheimer’s patients appear to be secondary to Wallerian degeneration of fibre due to neuronal loss in the association cortex [38, 39]. These changes are also a supportive finding that MDS-OAβ levels in the blood are related to changes in brain structure. However, the limitation of this study is that we could not suggest the severity and time sequence of the lesions correlated with MDS-OAβ in WM or GM. To get the answer, further studies are needed.

In this study, there was a good correlation between MDS-OAβ levels in the blood and GM and WM atrophy of the brain; however, it was not identified whether the Aβ oligomerization in the blood was related to the cause of brain atrophy in the form of AD. In order to add certainty, further longitudinal studies are to follow, and more specifically, studies on testing how brain MRI varies with baseline levels of MDS-OAβ in the blood are necessary. Furthermore, additional studies should be conducted on determining whether oligomerization of Aβ is dependent on health condition and whether it can affect brain structure.

## Conclusions

This VBM study found specific brain atrophy and its locations related to MDS-OAβ of plasma. The significant volume reduction was observed at the bilateral hippocampus, temporal, medial and dorsolateral parietal regions (FWE < 0.05) where typical AD pathologic changes are found. Higher MDS-OAβ could be related to AD brain degeneration, and this warrants further investigation.

## Additional files

Additional file 1: **Figure S1.** Grey matter volume reduction in the Alzheimer’s disease group (*n* = 39) compared to healthy normal control (*n* = 92) (corrected for age and total intracranial volume, family-wise error (FWE) < 0.01). (TIF 504 kb)

Additional file 2: **Figure S2.** The means of Multimer Detection System-Oligomeric Aβ of healthy normal control (HNC), subjective cognitive decline (SCD), mild cognitive impairment (MCI) and Alzheimer’s disease dementia (AD) (expressed by mean dot and standard error bar). (TIF 125 kb)
