# Supplementary figures and images for: Blood amyloid-β oligomerization associated with neurodegeneration of Alzheimer’s disease
Source: Alzheimers Res Ther. 2019 May 10;11:40. doi: 10.1186/s13195-019-0499-7 (PMC6511146; doi:10.1186/s13195-019-0499-7)

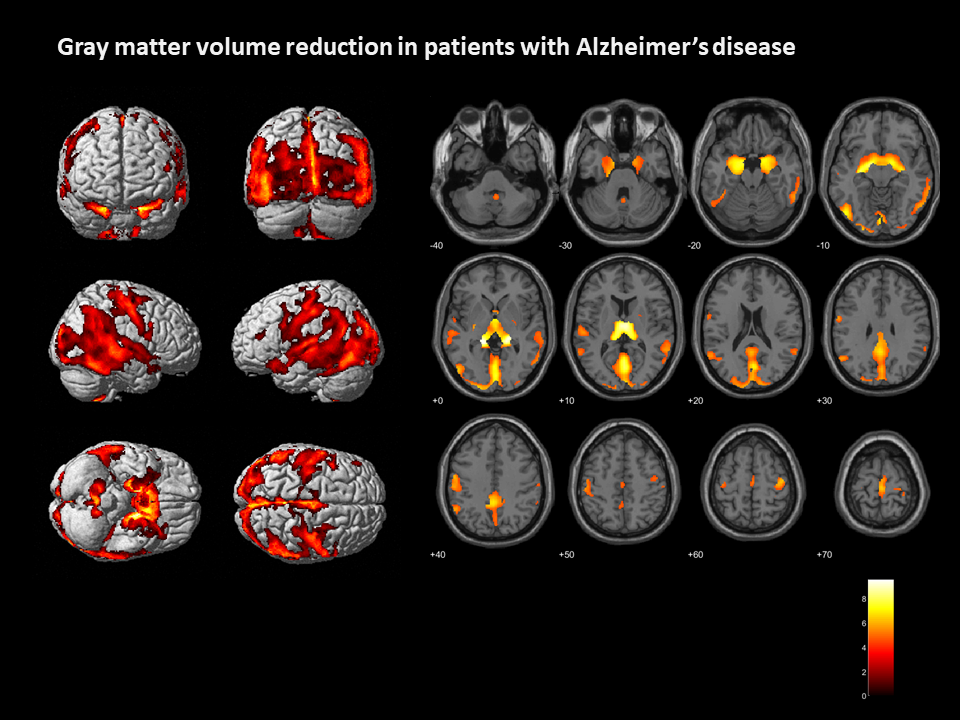

Supplement: Supplementary file 1 — Figure S1. Grey matter volume reduction in the Alzheimer’s disease group (n = 39) compared to healthy normal control (n = 92) (corrected for age and total intracranial volume, family-wise error (FWE) < 0.01). (TIF 504 kb) [file 13195_2019_499_MOESM1_ESM.tif]

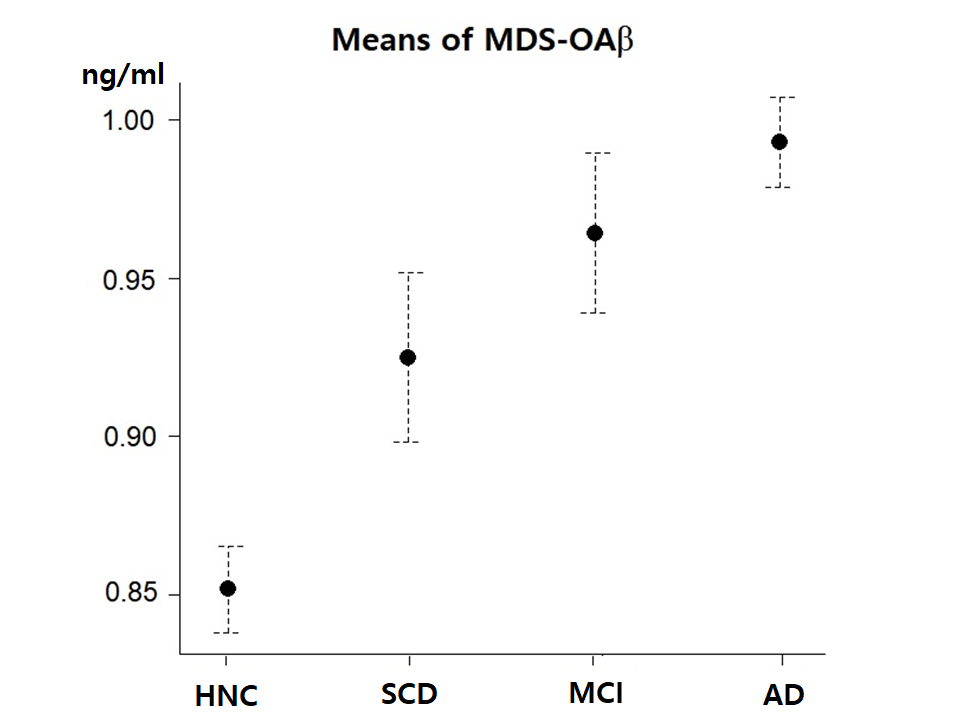

Supplement: Supplementary file 2 — Figure S2. The means of Multimer Detection System-Oligomeric Aβ of healthy normal control (HNC), subjective cognitive decline (SCD), mild cognitive impairment (MCI) and Alzheimer’s disease dementia (AD) (expressed by mean dot and standard error bar). (TIF 125 kb) [file 13195_2019_499_MOESM2_ESM.tif]
